# A Significant Association Between Rhein and Diabetic Nephropathy in Animals: A Systematic Review and Meta-Analysis

**DOI:** 10.3389/fphar.2019.01473

**Published:** 2019-12-13

**Authors:** Heng-Chang Hu, Liu-Tao Zheng, Hai-Yan Yin, Yuan Tao, Xiao-Qiong Luo, Kai-Shan Wei, Li-Ping Yin

**Affiliations:** ^1^Department of Endocrinology, Hospital of Chengdu University of Traditional Chinese Medicine, Chengdu, China; ^2^Department of Acupuncture, Chengdu University of Traditional Chinese Medicine, Chengdu, China

**Keywords:** rhein, diabetic nephropathy, traditional Chinese medicine, pharmacology, mechanism, meta-analysis

## Abstract

**Background:** Rhein is considered to have beneficial influence on diabetic nephropathy. Animal experiments suggested that the mechanisms of rhein against diabetic nephropathy may involve many processes, but the credibility of the evidence is unclear. Therefore, we conducted systematic review and meta-analysis of pre-clinical animal data to assess the current evidence for rhein effects and mechanisms in treating diabetic nephropathy.

**Methods:** The databases of PubMed, EMBASE, Web of Science, China National Knowledge Infrastructure, VIP information database, Wanfang Data Information Site, and Chinese Biomedical Literature were searched for this review. SYRCLE’s risk of bias tool for animal studies was applied to assess the methodological quality of studies. A meta-analysis was performed according to the Cochrane Handbook for Systematic Reviews of Interventions by using RevMan 5.3 and STATA/SE 12.0 software. This study was registered with PROSPERO, number CRD42018105220.

**Results:** Twenty-five studies involving 537 animals were included. There was significant association of rhein with levels of blood glucose (*P* < 0.05), serum creatinine (Scr) (*P* < 0.05), urine protein (*P* < 0.05), kidney tubules injury index (*P* < 0.05), relative area of kidney collagen (*P* < 0.05), transforming growth factor-β_1_ (*P* < 0.05), malondialdehyde (*P* < 0.05), and superoxide dismutase (*P* < 0.05) compared with that in the control group. No significant association between rhein and endothelin (*P* > 0.05) was found. Subgroup analysis showed that the hypoglycemic effect of rhein on type 2 diabetic nephropathy was better than on type 1 diabetic nephropathy (*P* < 0.05).

**Conclusions:** These findings suggested that rhein has beneficial effects on animal models of diabetic nephropathy, and that the mechanisms are mostly involved with ameliorating levels of TGF-β_1_, renal fibrosis, metabolism, and oxidative stress status. However, some factors such as possible publication bias, methodological quality, and sample size may affect the accuracy of positive findings. These limitations suggested that a cautious interpretation of the positive results of this systematic review and meta-analysis is necessary. Therefore, high methodological quality and well-reported animal experiments are needed in future research.

## Introduction 

Diabetic nephropathy (DN), one of the most common microvascular complications of diabetes (Brown, 2008), is the primary cause of chronic kidney disease (CKD) and end-stage renal disease (ESRD) worldwide (Skyler, 2001). Patients with DN are at substantially rising risk of cardiovascular events and mortality (Afkarian et al., 2013). Modern medical research has shown that diverse pathogenetic mechanisms account for the occurrence and development of DN, such as metabolic dysregulation, inflammatory mechanisms, oxidative stress, and abnormal cytokines (Badal and Danesh, 2014; Reidy et al., 2014). Because of multiple pathogenic mechanisms, multi-targeting therapy will likely be required for the development of effective therapeutics in DN. So traditional Chinese herb has received high attention in recent years due to its broad pharmacological effects. Therefore, to explore the feasible traditional Chinese herb is a hot topic in current research (Zeng et al.,2014).

Rhein (1,8-dihydroxy-3-carboxyl anthraquinone) is a monomer of anthraquinone compound mainly extracted from Chinese herb rhubarb, and the basic structure of anthraquinone nucleus is 1,8-dihydroxyanthracene (Xie, 2017; Zhang et al., 2017). The presence of substituent at the phenolic hydroxyl group can enhance antioxidant activity (Zong et al., 2008). There is also a lone pair of electrons on the phenolic hydroxyl group and carbonyl oxygen, so 1,8-phenolic hydroxyl group and 9-carbonyl group are suitable for coordination with various metal ions, which can exert corresponding pharmacological effects (Xiang, 2014). Nowadays, rhein is considered to have beneficial influence on DN. Some studies indicated that rhein can induce arrest in the mesangial cell cycle at the phase of G1, up-regulation the expression of apoptotic mediators of Bax and Caspase-3, which can promote mesangial cell apoptosis and alleviate renal fibrosis (Xu et al., 2016). In addition, rhein can down-regulation the expression of TGF-β_1_ and alleviate renal fibrosis. Liu et al. (2013) found that rhein could protect pancreatic B-cells from apoptosis by inhibiting the hyperglycemia-induced dynamin-related protein 1 (DRP1) expression and reduce blood glucose. Other therapeutic mechanisms such as anti-oxidative stress (Wang et al., 2011) and regulating related cytokines (Wang et al., 2012) were also reported. However, the therapeutic mechanisms of rhein for DN are still not completely clear, which is one of the main causes limiting rhein to clinical application. Although animal experiments suggested that the mechanisms of rhein against DN may be *via* many processes, it is difficult to draw definitive and reliable conclusions about the relevant mechanisms due to the small sample size and possible exaggerated efficacy of various individual animal experiments. Therefore, it is necessary to increase sample size and judge whether there are exaggerated intervention effects.

Systematic review and meta-analysis attempt to combine all empirical evidence from relevant studies to provide more precise estimates of the effects than those derived from individual studies and is always applied to evaluate the effectiveness of medicine (Higgins and Green, 2011). Therefore, we conducted systematic review and meta-analysis of pre-clinical animal data to assess the current evidence for rhein effects in treating DN. The purposes of this study were to (1) identify all animal experiments to illustrate the efficacy of rhein in animal models of DN, (2) provide precise empirical evidence of mechanisms associated with efficacy of rhein, (3) determine the appropriate conditions of rhein to enhance curative effects, (4) provide reference for clinical trials and clinical applications related to rhein, and (5) provide an evaluation of impact of possible publication bias and small-study effects.

## Methods

This meta-analysis was performed according to Cochrane Handbook for Systematic Reviews of Interventions (Higgins and Green, 2011). The protocol for this meta-analysis is available in PROSPERO (CRD42018105220).

### Search Strategies

The databases of PubMed, EMBASE, Web of Science, China National Knowledge Infrastructure (CNKI), VIP information database, Wanfang Data Information Site, and Chinese Biomedical Literature (CBM) were searched for this review with language restrictions to Chinese and English. Other restriction was imposed on publication time from January 2000 to July 2018. Search methods of MeSH terms with free words were applied in English databases. The related terms were as follows: Participants (Diabetic Nephropathies [MeSH], Diabetic Nephropathies, ‘Nephropathies, Diabetic’, ‘Nephropathy, Diabetic’, Diabetic Nephropathy, Diabetic Kidney Disease, Diabetic Kidney Diseases, ‘Kidney Disease, Diabetic’, ‘Kidney Diseases, Diabetic’, ‘DN’, ‘DKD’, Intervention (Rhein [MeSH], Rhein, Rheic Acid, Rheinic Acid, Rhubarb Extract, Extract of rhubarb, Monorhein, Cassic Acid, Rheochrysin, Parietic Acid). Chinese databases were searched with the aforementioned search terms in Chinese. In addition, some potentially relevant studies were identified through other approaches, such as conference literature and manual searching.

### Inclusion Criteria

(1)Participants: models of diabetic nephropathy (rats or mice); (2) Intervention: rhein with all dose and duration; (3) Control: purified water-treated, saline-treated, same solvent, or no treatment; (4) Outcomes: urine protein, blood glucose and serum creatinine (Scr) were the primary outcomes. Kidney tubules injury index, relative area of kidney collagen fiber, transforming growth factor-β_1_ (TGF-β_1_), malondialdehyde (MDA), superoxide dismutase (SOD), and endothelin (ET) were the secondary outcomes; (5) Study design: randomized controlled researches; (6) Language: Chinese and English.

### Exclusion Criteria

(1)Participants: *in vitro* studies and researches in humans; (2) Intervention: rhein without batch number; (3) Control: other Chinese herbal medicine and rhein analogue (emodin, etc.); (4) Study design: case reports, cross-over studies and studies without a separate control group; (5) Pilot studies; (6) Reviews; (7) Duplicate publication; (8) Studies without full-text.

### Data Collection

Two reviewers extracted the following items independently from included studies: (1) Study ID: name of the authors and year of publication; (2) Information of participants: number, weight, gender, species, and models of DN in the experiment group and the control group; (3) Information of treatment: dosage and duration; (4) Outcome measures: urine protein, blood glucose, Scr, kidney tubules injury index, relative area of kidney collagen fiber, TGF-β_1_, MDA, SOD, and ET. All the outcome measures were continuous variables, so each variable was extracted and expressed as values of mean and standard deviation from each treatment and control group of all studies. If the outcomes were presented at different time points, each variable was extracted from the last time point. For studies with more than one experiment groups sharing one control group, we separated this control group into multiple groups (the number was the same as experiment groups) and incorporated these comparisons into this meta-analysis (Higgins and Green, 2011). Reviewers would discuss with a third reviewer if they had different views on the same point.

Reviewers would send an email to authors of related studies for more details if it was necessary. In addition, when the items of studies and data of outcomes were missing or only expressed graphically, a request would be sent to authors for more information. Qualitative analysis would be used if relevant data was not available.

### Quality Assessment

SYRCLE’s risk of bias tool for animal studies was applied to assess the methodological quality of studies (Hooijmans et al., 2014): (1) sequence generation; (2) baseline characteristics; (3) allocation concealment; (4) random housing; (5) blinding (performance bias); (6) random outcome assessment; (7) blinding (detection bias); (8) incomplete outcome data; (9) selective outcome reporting; (10) other sources of bias.

Two reviewers performed quality assessment independently, any disagreements of them would be discussed with a third reviewer.

### Statistical Analysis

According to the Cochrane Handbook for Systematic Reviews of Interventions (Higgins and Green, 2011), standardized mean difference (SMD) was considered to express the pooled effect sizes for continuous outcomes (e.g. urine protein, blood glucose). The confidence interval (CI) was established at 95%, and *P* value < 0.05 was considered to be statistically significant. Random effect model was utilized to calculate the pooled results because this model incorporated between-study variability and provided more conservative pooled estimates (Becic and Studenik, 2018). The Chi-square test with a significance level of α = 0.1 was used as statistical measure of heterogeneity between the different studies. The *I* ^2^ statistic was applied to quantify inconsistency between studies, where *I* ^2^ statistic of 50% or more indicated a considerable heterogeneity. Subgroup analysis was conducted to investigate the sources of heterogeneity and its influence on effect size according to following factors if there were adequate studies: duration (< 12weeks, ≥12weeks), dosage (low < 50mg, 50≤medium < 100mg, high≥100mg), models of DN (type 1 DN, type 2 DN), species (rats, mice). Sensitivity analysis was performed to explore the impact of an individual study by omitting studies one by one if there were sufficient studies. Publication bias and small-study effects were evaluated by a funnel plot and Egger’s test (Egger, 1997) if there were at least 10 studies for each outcome. For Egger’s test, *P* value of greater than 0.05 was determined as no considerable publication bias or small-study effects in studies (Egger et al., 1997). Meta-analysis and subgroup analysis were performed with RevMan V.5.3 software. Sensitivity analysis and Egger’s test were performed by using STATA/SE 12.0 software.

## Results

### Study Inclusion

**Figure 1:**
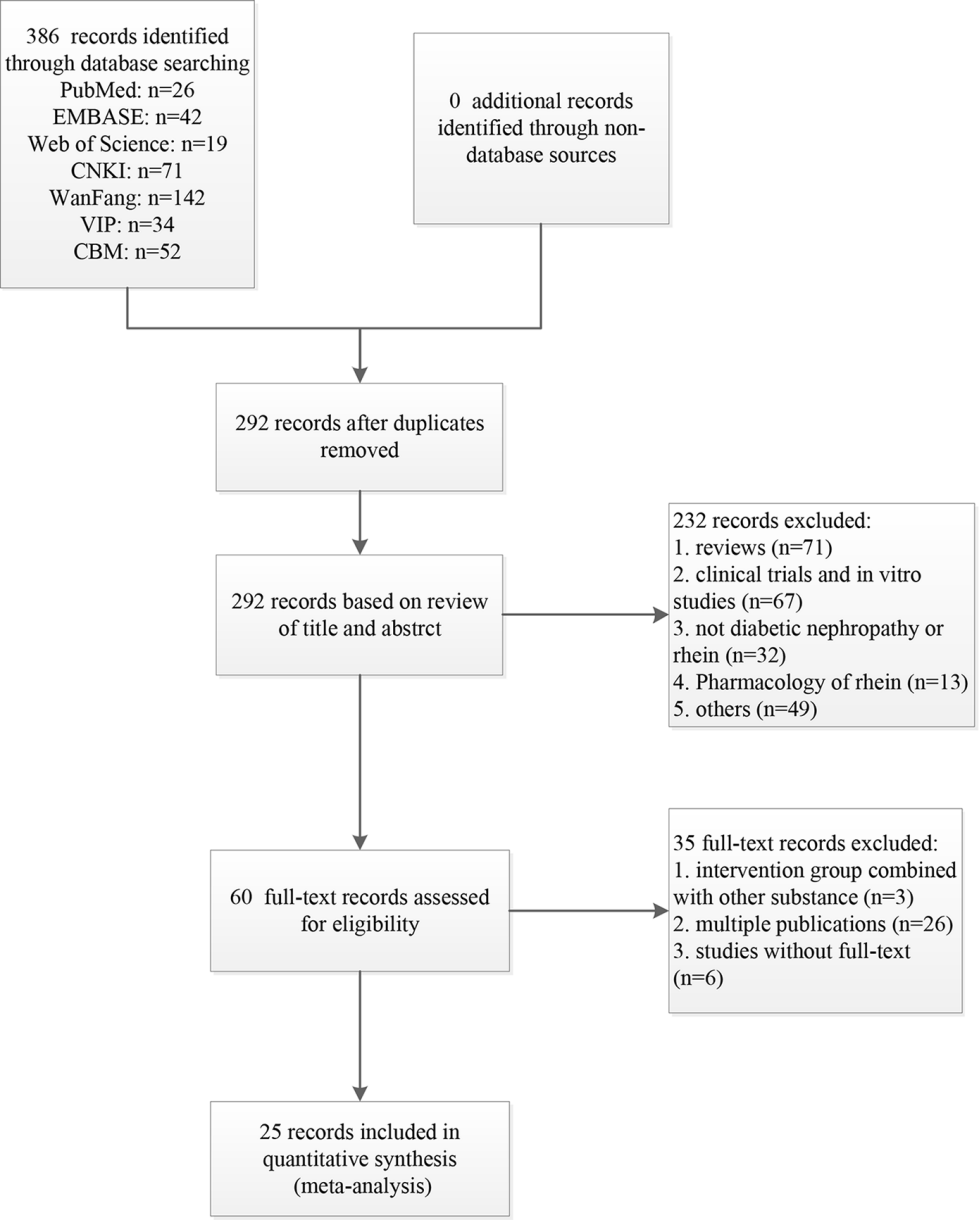
Flow diagram of the study selection process for this systematic review and meta-analysis.

A total of 386 records were identified from the searches for systematic review and meta-analysis. After removing duplicates, 292 records remained. Based on titles and abstracts, 232 records were excluded due to the following reasons: (1) reviews; (2) clinical trials or *in vitro* studies; (3) not diabetic nephropathy or rhein; (4) pharmacology of rhein. (5) others. The 60 remaining records were downloaded for further selection. Three records were removed because intervention group combined with other substance, 26 records were excluded because of multiple publication, and 6 records were excluded due to the deficiency of full-text. Ultimately, 25 eligible studies were identified (**Figure 1**).

### Study Characteristics

The 25 studies involving 537 animals were ultimately included in this systematic review and meta-analysis. The number of all animals in the intervention group was 300 and that in the control group was 237. The species of animals included rats and mice, 16 studies (64%) used rats and 9 studies (36%) used mice. The weight of rats ranged from 150 to 250 g in all studies and that of mice was not reported in related studies. Nine out of 25 studies (36%) used diabetic nephropathy models with type 2 DN, 8 studies (32%) utilized diabetic nephropathy models with type 1 DN, and the rest studies (32%) did not report the type of animal models.

Three levels (high, medium, and low) of dosage of rhein were used in this study. The high dose was used most commonly in experiment, 17 out of 25 studies (68%) used high dose. The medium dose was applied in 3 studies (12%), and low dose was utilized least. Five studies contained two different dosages simultaneously. The control group mainly included carboxymethyl cellulose (CMC) and saline. Fourteen studies (56%) utilized saline. Five studies (20%) used CMC, but one out of these five studies did not report the concentration of CMC, the rest 4 studies reported that the concentration of CMC was 0.5%. One study (4%) used 0.5% CMC with saline, and 2 studies (8%) selected water as the control intervention. The duration consisted of short and long duration. Fifteen studies (60%) selected short duration and 8 studies (32%) used long duration, the rest studies included both long and short course. The characteristics of the 25 included studies were shown in **Table 1** (Guo et al., 2002; Zhu et al., 2002; Ai et al., 2004; Huang et al., 2004; Zhang et al., 2005; Gong et al., 2006; Jia et al., 2007; Liu and Ning, 2007; Jin et al., 2008; Li, 2008; Gao et al., 2010; Li and Yang, 2010; Wang et al., 2011; Huang et al., 2012; Wang et al., 2012; Chen et al., 2013; Hu et al., 2014; Chen et al., 2015a; Chen et al., 2015b; Duan et al., 2015; Wu, 2015; Li and Zhen, 2017; Lin et al., 2017; Qiao et al., 2017; Zhang et al., 2017).

**Table 1:** Characteristics of the included studies.

Study year	n = (E, C)	Gender	Species	Weight (g)	Model method	Rhein dose (mg/kg)	Duration (weeks)	Control	Outcome index
Ai et al., 2004	9, 8	male	rat	150-190	STZ (50mg/kg)	70	12	saline	1.Urine protein 2. Blood glucose 3.ET
Chen et al., 2015a	6, 6	male	rat	170-200	STZ (50mg/kg)	150	8	saline	1.Blood glucose 2.Scr
Chen et al., 2015a	8, 8	male	rat	180-220	high fat-sugar diet 8 weeks and STZ (25mg/kg)	80	8	saline	1.Urine protein 2. Blood glucose 3.TGF-β1 mRNA
Chen et al., 2015b	10/11, 10	male	rat	180-220	high fat-sugar diet 4 weeks, STZ (35mg/kg)	50 100 150	10	saline	1.urine protein 2. blood glucose 3.Scr
Duan et al., 2015	6, 6	male/ female	mice	NR	NR	120	12	5%CMC	1.Blood glucose 2.Scr
Gao et al., 2010	6, 6	male/ female	mice	NR	NR	150	12	saline	1.Blood glucose 2.Scr
Gong et al., 2006	24/24, 24	female	rat	180-200	STZ (20mg/kg) 3 times and STZ (10mg/kg) 1 time	35 70	12	5%CMC	1.urine protein 2. blood glucose 3.Scr
Guo et al., 2002	10, 10	female	rat	180	high fat-sugar diet 1 month and STZ (25mg/kg)	100	24	0.5% CMC+ saline	1.Urine protein 2. Blood glucose
Hu et al., 2014	20, 20	male	rat	200-240	high fat-sugar diet 2 weeks and STZ (35mg/kg)	100	8	saline	1.Blood glucose 2.Scr 3.Urine protein
Huang et al., 2004	6, 6	male/ female	mice	NR	NR	120	12	saline	Blood glucose
Huang et al.,2012	15, 15	male	rat	180-200	NR	100	6	purified water	1.Blood glucose 2. Scr 3.MDA 4.SOD
Jia et al., 2007	6, 6	male/ female	mice	NR	NR	150	12	saline	1.Blood glucose 2.Scr
Jin et al., 2008	6, 6	male	rat	NR	STZ (55mg/kg)	100	8 16	CMC	1.Urine protein 2.Scr 3.Kidney tubules injury index 4.Relative area of kidney collagen fiber
Li, 2008	6, 6	male	rat	220-250	STZ (55mg/kg)	100	8 16	saline	1.Urine protein 2.Scr 3.Kidneytubules injury Index 4. Relative area of kidney collagen fiber
Li and Yang, 2010	8, 8	male	rat	200-210	STZ (65mg/kg)	100	10	0.5% CMC	1.Urine protein 2.Blood glucose
Li and Zhen, 2017	6, 6	NR	mice	NR	NR	25 50	12	saline	1.Blood glucose 2. Scr 3.MDA 4.SOD
Lin et al., 2017	12/12, 12	male	mice	NR	STZ (50mg/kg)	25 50	15	NR	1. Blood glucose 2.Scr 3.MDA 4.SOD
Liu and Ning,2007	20, 20	male	rat	220-250	STZ (60mg/kg)	150	16	water	1. Urine protein 2. Blood glucose 3. Kidney tubules injury index 4. Relative area of kidney collagen fiber
Qiao et al., 2017	8/8/8, 6	male/ female	rat	160-180	high fat-sugar diet 4weeks and STZ (35mg/kg)	50 100 150	8	saline	1. Blood glucose 2.Scr
Wang et al., 2011	8, 8	male	rat	NR	high fat-sugar diet 8 weeks and STZ (25mg/kg)	100	8	saline	1. Urine protein 2. Blood glucose 3.MDA 4.SOD
Wang et al., 2012	8, 9	male/ female	rat	150-180	STZ (40mg/kg)	70	12	saline	1. Urine protein 2. Blood glucose 3.ET
Wu, 2015	6, 6	male	mice	NR	NR	120	12	5% CMC	1. Blood glucose 2. Scr
Zhang et al., 2005	9, 9	female	rat	178-196	high fat-sugar diet 3 month and STZ (25mg/kg)	100	12	NR	1. Urine protein 2. Blood glucose 3.TGF-β1 mRNA
Zhang et al., 2017	6, 8	male	mice	NR	0.2% adenine containing diet feeding	120	12	NR	Scr
Zhu et al., 2002	8, 8	male/ female	mice	NR	NR	120	12	saline	1. Blood glucose 2.Scr

NR, not report; STZ, streptozotocin; CMC, carboxymethyl cellulose.

### Study Quality

Random allocation to the control group and the intervention group were mentioned in 19 studies (76%), of which two studies described an adequate random sequence generation process (random number table method). Nine studies (36%) reported the baseline characteristics of animals. None of the studies mentioned the application of allocation concealment. No study described random housing, blinding (performance bias), and random outcome assessment. Blinding (detection bias) was not mentioned in all studies. All studies had complete outcome data and reported expected outcomes. There were no other sources of bias existed in all studies. The methodological quality of each study was shown in **Table 2**.

**Table 2:** Risk of bias of included studies.

Study year	(1)	(2)	(3)	(4)	(5)	(6)	(7)	(8)	(9)	(10)
Ai et al., 2004	U	U	U	U	U	U	Y	Y	Y	Y
Chen et al.,2013	N	U	U	U	U	U	Y	Y	Y	Y
Chen et al., 2015b	Y	U	U	U	U	U	Y	Y	Y	Y
Chen et al., 2015a	U	U	U	U	U	U	Y	Y	Y	Y
Duan et al., 2015	U	Y	U	U	U	U	Y	Y	Y	Y
Gao et al., 2010	U	Y	U	U	U	U	Y	Y	Y	Y
Gong et al., 2006	U	Y	U	U	U	U	Y	Y	Y	Y
Guo et al., 2002	U	U	U	U	U	U	Y	Y	Y	Y
Hu et al., 2014	Y	U	U	U	U	U	Y	Y	Y	Y
Huang et al., 2004	U	U	U	U	U	U	Y	Y	Y	Y
Huang et al.,2012	U	Y	U	U	U	U	Y	Y	Y	Y
Jia et al., 2007	U	Y	U	U	U	U	Y	Y	Y	Y
Jin et al., 2008	U	U	U	U	U	U	Y	Y	Y	Y
Li, 2008	U	U	U	U	U	U	Y	Y	Y	Y
Li and Yang, 2010	U	U	U	U	U	U	Y	Y	Y	Y
Li and Zhen, 2017	N	U	U	U	U	U	Y	Y	Y	Y
Lin et al., 2017	N	U	U	U	U	U	Y	Y	Y	Y
Liu and Ning, 2007	U	U	U	U	U	U	Y	Y	Y	Y
Qiao et al., 2017	U	Y	U	U	U	U	Y	Y	Y	Y
Wang et al., 2011	U	U	U	U	U	U	Y	Y	Y	Y
Wang et al., 2012	U	U	U	U	U	U	Y	Y	Y	Y
Wu, 2015	U	Y	U	U	U	U	Y	Y	Y	Y
Zhang et al., 2005	N	Y	U	U	U	U	Y	Y	Y	Y
Zhang et al., 2017	N	U	U	U	U	U	Y	Y	Y	Y
Zhu et al., 2002	N	Y	U	U	U	U	Y	Y	Y	Y

(1) Sequence generation; (2) Baseline characteristics; (3) Allocation concealment; (4) Random housing; (5) Blinding (performance bias); (6) Random outcome assessment; (7) Blinding (detection bias); (8) Incomplete outcome data; (9) Selective outcome reporting; (10) Other sources of bias; Y: yes; N: no; U: unclear.

### Effectiveness

#### Blood Glucose

**Figure 2:**
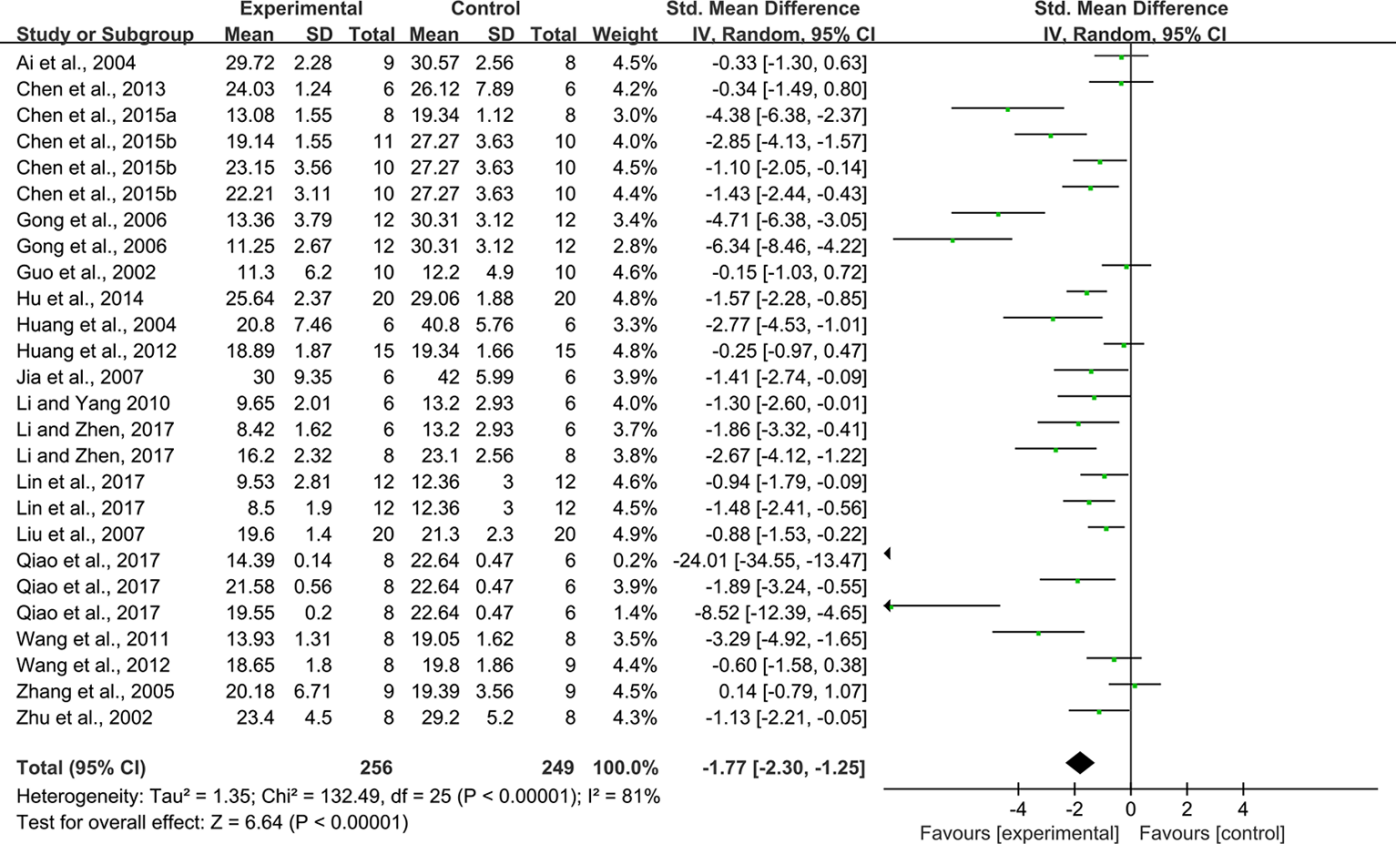
Pooled estimate of blood glucose with rhein.

Twenty-six studies (some studies contain multiple intervention groups) reported the impact of rhein on this outcome. The pooled analysis showed significant effect of rhein for reducing blood glucose compared with that in the control group (n = 505, SMD = -1.77, 95% CI [-2.30, -1.25], *P* < 0.00001; Heterogeneity: Chi² = 132.49, *P* < 0.00001, *I*² = 81% **Figure 2**).

#### Scr

**Figure 3:**
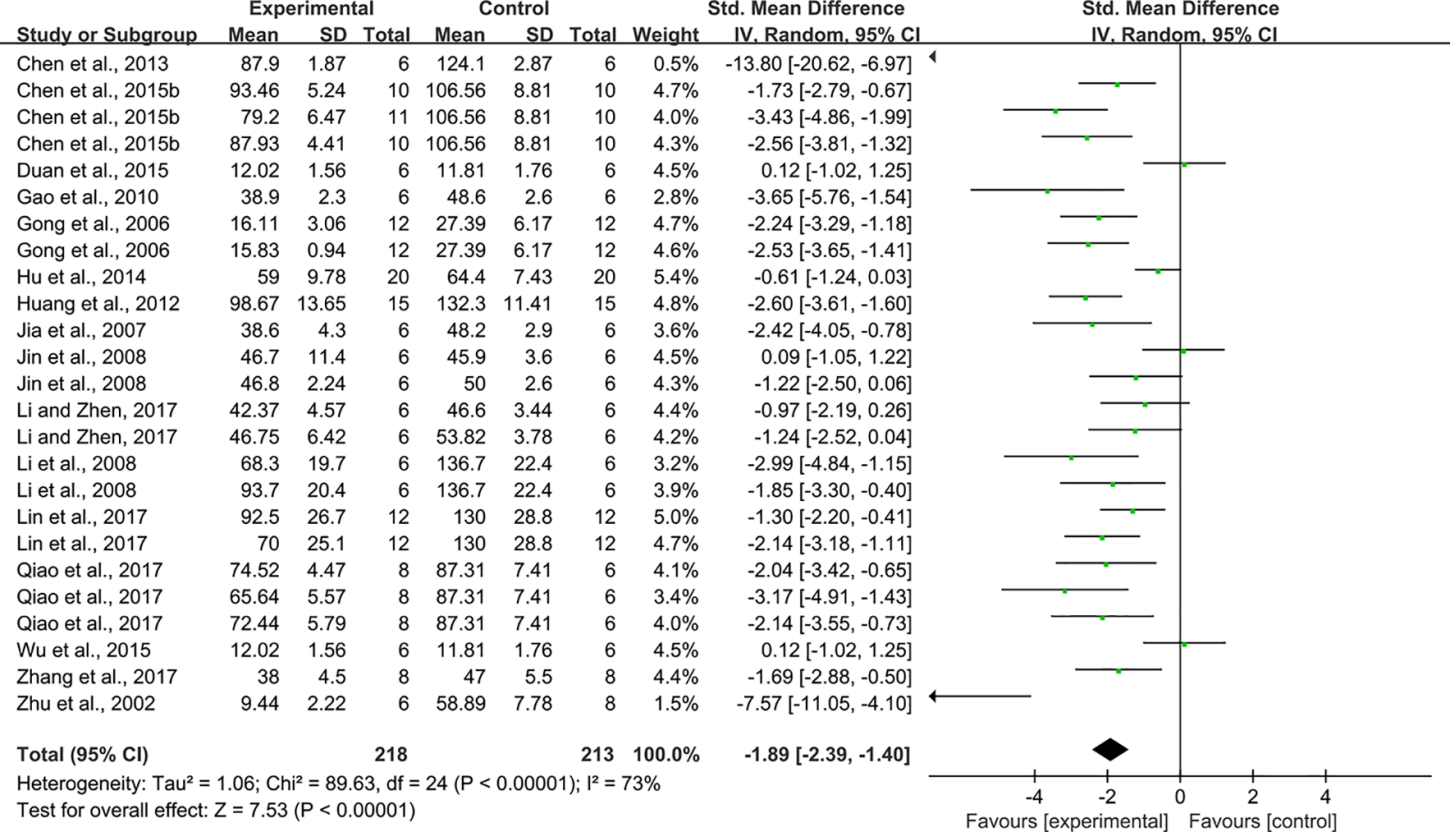
Pooled estimate of Scr with rhein.

Twenty-five studies reported the impact of rhein on Scr. The pooled result showed that rhein significantly decreased Scr compared with that in the control group (n = 431, SMD = -1.89, 95% CI [-2.39, -1.40], *P* < 0.00001; Heterogeneity: Chi² = 89.63, *P* < 0.00001; *I*² = 73% **Figure 3**).

#### Urine Protein

**Figure 4:**
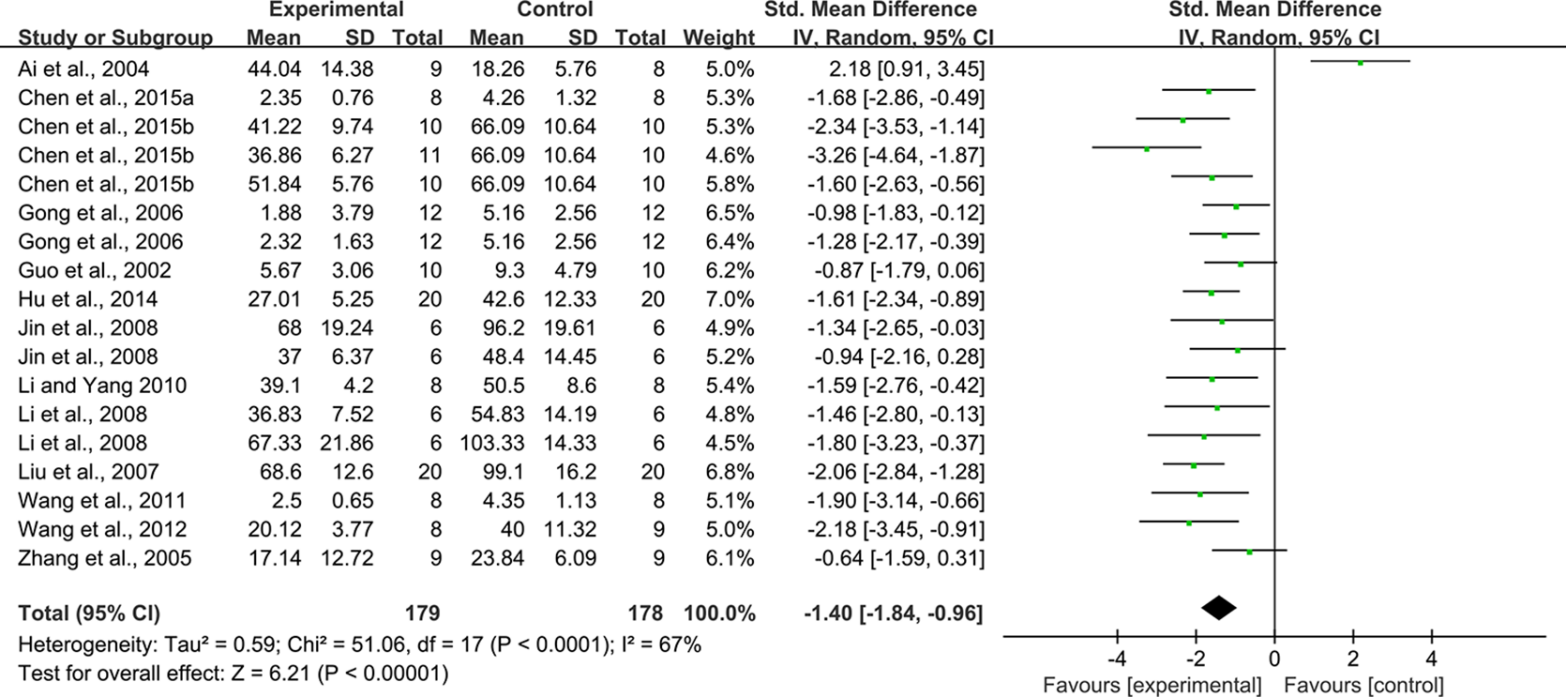
Pooled estimate of urine protein with rhein.

With regard to effect on urine protein, 18 studies reported on this outcome. The pooled result showed that rhein significantly decreased urine protein compared with that in the control group (n = 357, SMD = -1.40, 95% CI [-1.84, -0.96], *P* < 0.0001; Heterogeneity: Chi² = 51.06, *P* < 0.0001, *I*² = 67% **Figure 4**).

#### Kidney Tubules Injury Index

**Figure 5:**
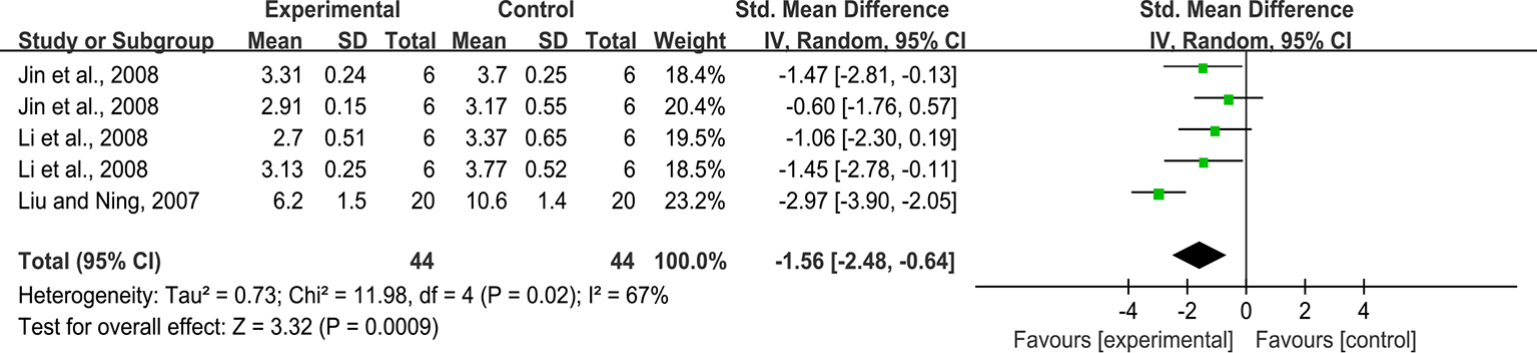
Pooled estimate of kidney tubules injury index with rhein.

Five studies reported the impact of rhein on this outcome. The pooled result showed that rhein significantly decreased kidney tubules injury index compared with that in the control group (n = 88, SMD = -1.56, 95% CI [-2.48, -0.64], *P* = 0.0009; Heterogeneity: Chi² = 11.98, *P* = 0.02, *I*² = 67% **Figure 5**).

#### Relative Area of Kidney Collagen Fiber

**Figure 6:**
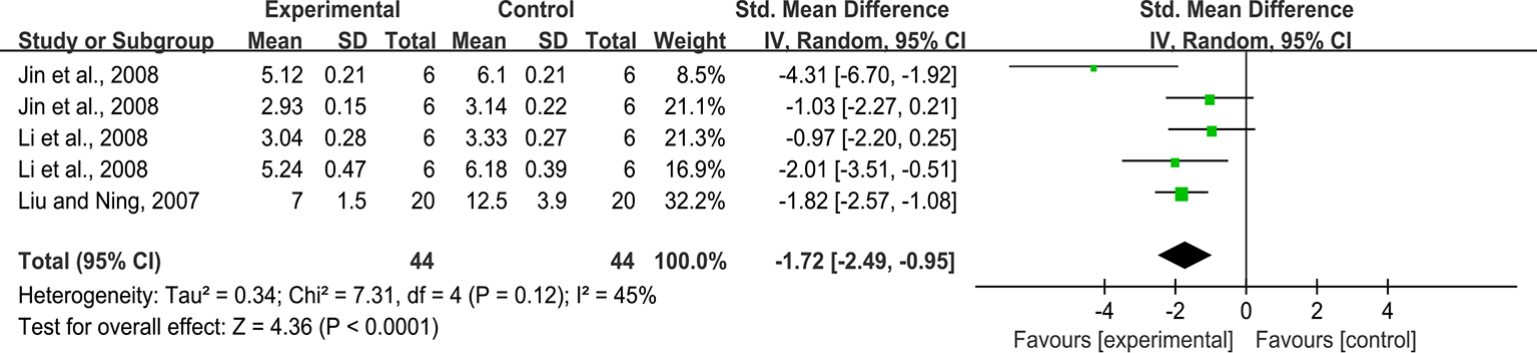
Pooled estimate of relative area of kidney collagen fiber with rhein.

Five studies reported on this outcome. The pooled result showed that rhein significantly decreased relative area of kidney collagen fiber compared with that in the control group (n = 88, SMD = -1.72, 95% CI [-2.49, -0.95], *P* < 0.0001; Heterogeneity: Chi² = 7.31, *P* = 0.12; *I* ^2^ = 45% **Figure 6**).

#### MDA

**Figure 7:**
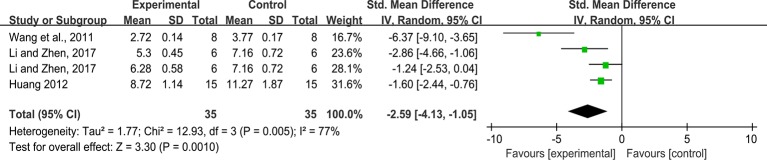
Pooled estimate of MDA with rhein.

As for the effect on MDA, four studies reported the impact of rhein on this outcome. The pooled result showed rhein significantly decreased MDA compared with that in the control group (n = 70, SMD = -2.59, 95%,CI [-4.13, -1.05], *P* = 0.001; Heterogeneity: Chi² = 12.93, *P* = 0.005, *I*² = 77% **Figure 7**).

#### SOD

**Figure 8:**
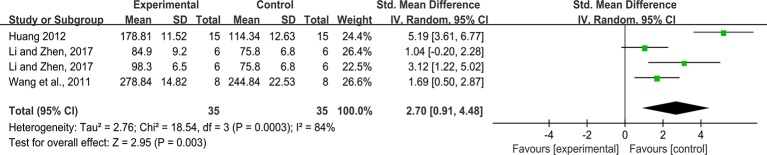
Pooled estimate of SOD with rhein.

Effect sizes for SOD were pooled from a total of 4 studies. There was significant association of rhein with SOD (n = 70, SMD = 2.70, 95%CI [0.91, 4.48], *P* = 0.003; Heterogeneity: Chi² = 18.54, *P* = 0.0003, *I*² = 84% **Figure 8**).

#### ET

**Figure 9:**
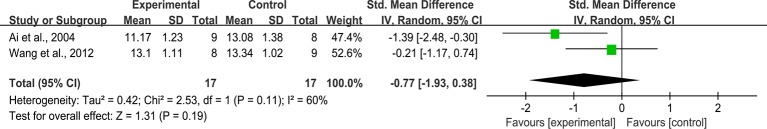
Pooled estimate of ET with rhein.

Two studies reported on this outcome. The pooled result showed that rhein did not significantly decreased ET compared with that in the control group (n = 34, SMD = -0.77, 95%CI [-1.93, 0.38], *P* = 0.19; Heterogeneity: Chi² = 2.53, *P* = 0.11; *I* ^2^ = 60% **Figure 9**).

#### TGF-β_1_

**Figure 10:**
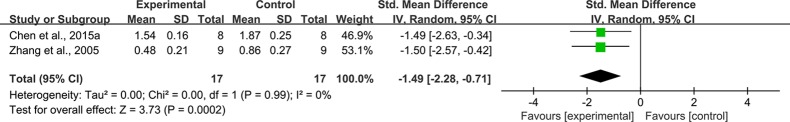
Pooled estimate of TGF-β_1_ with rhein.

Effect sizes for TGF-β_1_ were pooled from a total of 2 studies. There was significant association of rhein with TGF-β_1_ (n = 34, SMD = -1.49, 95%CI [-2.28, -0.71], *P* = 0.0002; Heterogeneity: Chi² = 0.00, *P* = 0.99, *I*² = 0% **Figure 10**).

There were no data on blood glucose, MDA and SOD in these studies listed as follows: Duan et al., 2015; Gao et al., 2010; Wu, 2015; Lin et al., 2017, but blood glucose, MDA and SOD were presented graphically. These studies were not included in quantitative analyses, but all reported that rhein had significant effects on blood glucose (*P* < 0.05), MDA (*P* < 0.05) and SOD (*P* < 0.05).

### Subgroup Analysis

(1) Blood glucose. There was no significant difference between the subgroups of duration (*P* = 0.42), dosage (*P* = 0.73), and species (*P* = 0.15). As for models of animals, the efficacy of rhein on type 2 DN was better than on type 1 DN (*P* = 0.01) (**Table 3**).

(2) Scr. There was no significant association of rhein with Scr in subgroups for duration (*P* = 0.48), dosage (*P* = 0.53), models of animals (*P* = 0.17), and species (*P* = 0.97) (**Table 4**).

(3) Urine protein. There was no significant difference in effect sizes relative to duration (*P* = 0.16), dosage (*P* = 0.32), and models of animals (*P* = 0.52) (**Table 5**).

However, subgroup analysis of kidney tubules injury index, relative area of kidney collagen fiber, MDA, SOD, ET, and TGF-β_1_ were not conducted because there were no sufficient studies.

**Table 3:** Subgroup analysis according to blood glucose.

Variables	No. of studies	No. of participants	SMD [95%CI]	*P* value
Duration				
< 12 weeks	13	251	-1.59 [-2.25, -0.93]	0.42
?12 weeks	13	254	-2.04 [-2.91, -1.17]	
Dosage				
High dose	15	301	-1.59 [-2.27, -0.90]	0.73
Medium dose	8	60	-1.98 [-2.98, -0.98]	
Low dose	3	144	-2.22 [-4.24, -0.19]	
Models of animals				
Type 1 DN	7	150	-0.94 [-1.40, -0.48]	0.01
Type 2 DN	14	291	-2.56 [-3.55, -1.57]	
NR	5	64	-1.53 [-2.13, -0.94]	
Species				
Rat	19	393	-1.97 [-2.68, -1.27]	0.15
Mice	7	112	-1.37 [-1.80, -0.94]	

(NR, not report; P value, P, value for test for subgroup difference. the same below).

**Table 4:** Subgroup analysis according to Scr.

Variables	No. of studies	No. of participants	SMD [95%CI]	*P* value
Duration				
< 12 weeks	11	209	-2.11 [-2.87, -1.36]	0.48
?12 weeks	14	222	-1.74 [-2.42, -1.06]	
Dosage				
High dose	17	277	-1.89 [-2.61, -1.17]	0.53
Medium dose	5	94	-2.18 [-2.72, -1.64]	
Low dose	3	60	-1.71 [-2.34, -1.10]	
Models of animals				
Type 1 DN	7	108	-1.35 [-2.27, -0.43]	0.17
Type 2 DN	11	235	-2.40 [-3.11, -1.69]	
NR	7	88	-1.60 [-2.66, -0.55]	
Species				
Rat	15	281	-1.92 [-2.53, -1.31]	0.97
Mice	10	150	-1.90 [-2.78, -1.01]	

**Table 5:** Subgroup analysis according to urine protein.

Variables	No. of studies	No. of participants	SMD [95%CI]	*P* value
Duration				
< 12 weeks	10	191	-1.66 [-2.05, -1.27]	0.16
?12 weeks	8	166	-1.01 [-1.84, -0.18]	
Dosage				
High dose	12	239	-1.60 [-1.98, -1.22]	0.32
Medium dose	5	94	-0.93 [-2.30, 0.45]	
Low dose	1	24	-0.98 [-1.83, -0.12]	
Models of animals				
Type 1 DN	8	138	-1.16 [-2.11, -0.21]	0.52
Type 2 DN	10	219	-1.50 [-1.91, -1.09]	

### Sensitivity Analysis

1. Blood glucose. A sensitivity analysis was performed by separately excluding any one study and the results did not alter radically (**Figure S1**).
2. Scr. A sensitivity analysis was conducted by separately excluding any one study and the results did not change substantially (**Figure S2**).
3. Urine protein. Sensitivity analysis showed that the results did not change radically after removing any one study (**Figure S3**).

There was no sensitivity analysis of kidney tubules injury index, relative area of kidney collagen fiber, MDA, SOD, ET, and TGF-β_1_ because of insufficient studies.

### Publication Bias

1. Blood glucose. Visual inspection of funnel plots showed asymmetry for the effect of rhein on blood glucose (**Figure S4**), while the result of Egger’ s test was statistically significant: -5.01, 95% CI [-6.52, -3.50], *P* = 0.000 (**Figure S5**).
2. Scr. Visual inspection of funnel plots showed asymmetry for the effect of rhein on Scr (**Figure S6**), while this result was supported by Egger’s test: -3.95, 95% CI [-5.79, -2.10], *P* = 0.000 (**Figure S7**).
3. Urine protein. The funnel plot of 18 studies was basically asymmetric for the effect of rhein on urine protein (**Figure S8**), but this result was not supported by Egger’s test: -0.08, 95% CI [-4.36, 4.20], *P* = 0.97 (**Figure S9**).

Assessment of publication bias was not conducted in other outcome measures because less than 10 studies were included.

## Discussion

### Efficacy of Rhein

This meta-analysis intended to determine whether the rhein exerted effects on preventing DN in animal models. The results of meta-analysis indicated that rhein was significantly associated with lower levels of blood glucose, Scr and urine protein in animal models of DN. Sensitivity analysis that excluded one study each time did not alter these results. The mechanisms of efficacy of rhein mostly involved with enhancing SOD and decreasing TGF-β_1_, MDA, kidney tubules injury index, and relative area of kidney collagen fiber.

### Implication for Further Studies

The main risk factors for the development or progression of DN are poor glycemic control, and a mass of studies suggested that controlling glucose to nearly normal levels can reduce the urinary albumin excretion rate (UAER) and prevent the progression to overt proteinuria (Skyler, 2001). So, improving blood glucose plays an effective role in renal protection. For effect of rhein on glucose, some studies found that rhein did not decrease the level of blood glucose in the animal models of DN (Ai et al., 2004; Chen et al., 2013). Results of this study showed that rhein was associated with a lower level of blood glucose, and subgroup analysis indicated that there was a certain relationship between the hypoglycemic effect and the models of DN, a better hypoglycemic effect to rhein on type 2 DN than on type 1 DN (*P* < 0.05). Given their different pathogenic mechanisms, type 2 DN rooted in type 2 diabetic mellitus (DM) which was mainly dominated by insulin resistance, while type 1 DN rooted in type 1 DM which was caused by absolute absence of insulin. It is obvious that the insulin resistance was the main difference between type 1 DN and type 2 DN. Gao et al. (2010) found that rhein had an ameliorating effect on insulin resistance and decreased hyperglycemia mainly by attenuating insulin resistance. Thus, the capacity of rhein to ameliorate insulin resistance might be the reason why it had a more powerful hypoglycemic effect on type 2 DN. In clinical applications, it is particularly necessary to ascertain whether the patients are with type 1 DN or type 2 DN. During the treatment, clinician must pay attention to the changes of blood glucose, hemoglobin, and insulin in patients with different types of DN, and determine which type of DN is more suitable for the treatment of rhein.

Scr is one of the most important parameters which reflected renal function status. Duan et al. (2015) indicated that the level of Scr exhibited no considerable difference between rhein-treated group and control group. A number of studies suggested that rhein could decrease Scr, but it did not reach statistical significance (Hu et al., 2014; Li and Zhen, 2017). In this study, the results found that rhein had significant impact in decreasing Scr, which suggested that rhein could improve renal function of animals with DN. Urine protein would correspondingly decrease with the recovery of renal function. In this study, it was observed that the urine protein in the rhein-treated group was lower than that in the control group.

Dose-response relationship and time-effect relationship play a substantial role in clinical medication. Thus, whether the dosage and duration of rhein influence its intervention effects should be investigated. Subgroup analysis revealed that variability in dosage and duration were not associated with levels of blood glucose (*P* > 0.05), Scr (*P* > 0.05), and urine protein (*P* > 0.05). Based on these results, there might have been no dose-response relationship and time-effect relationship, or the dose and duration might have all been large or long enough to produce maximal responses in these experiments. In clinical applications, attention should be paid to the dosage and duration of rhein. It is necessary to note that the dosage of rhein should be increased slowly from an initiation of small dose in clinical medication. In the process of increasing the dose of rhein, it is absolutely essential to periodically detect the relevant indicators of DN to determine the optimal dosage. In addition, the medication duration is also very significant. During the treatment, it is important to focus on whether the treatment effect will get better with extension of time, or achieve the optimal effect at a certain period.

In the kidney, TGF-β_1_ is the most highly expressed, especially in tubular epithelial cells (Sharma and Ziyadeh, 1995). Emerging evidence suggested that TGF-β_1_ is a major cytokine mediated the occurrence of renal fibrosis and it is associated with the degree of renal fibrosis in DN (Fuad et al., 2000). TGF-β_1_ is seen in almost all patients with DN. In 2005, it was reported that the level of TGF-β_1_ in the placebo group increased by 43% from baseline after 1 year, while there was no significant increase in TGF-β_1_ level occurred in the ruboxistaurin-treated group by a phase II randomized controlled trial in 123 patients with type 2 DM and persistent albuminuria (Gilbert et al., 2007). Guo et al. (2002) found that rhein could reduce the accumulation of extracellular matrix (ECM) in the renal tubular epithelial induced by TGF-β_1_ and expression of collagen. Additional research (Jia et al., 2007) found that rhein could inhibit TGF-β_1_ expression. In this meta-analysis, the levels of TGF-β_1_, kidney tubules injury index and relative area of kidney collagen fiber were markedly reduced in the treatment group, which further confirmed that rhein could suppress TGF-β_1_ expression and ameliorate the degree of renal fibrosis through decreasing level of TGF-β_1_.

Oxidative stress is of great effect for the occurrence and development of DN, while SOD and MDA are common indicators of oxidative stress. Lin et al. (2017) indicated that rhein increased the level of SOD and decreased the level of MDA in kidney tissues of KK/HlJ mice. (Li and Zhen, 2017) found that medium-dose rhein had stronger effect on ameliorating SOD and MDA than low-dose. Results reported here suggested that rhein could decrease the level of MDA and increase the activity of SOD. However, there were no sufficient studies to conduct subgroup analysis to determine whether there was a dose-response relationship between rhein and SOD, MDA, so relevant studies of dose-response relationship should be carried out in the future.

ET is an endogenous vasoactive bioactive peptide which has great effects on renal function such as renal plasma flow (RPF), glomerular filtration rate (GFR), and concentration and dilution of urine (Simonson, 1993). Modern experiments noted that increased synthesis and secretion of renal ET may be associated with the occurrence of DN. Ai et al. (2004) reported that rhein was significantly associated with the lower level of ET. Results reported here showed that rhein was not associated with the lower level of ET (*P* = 0.21). It is possible that rhein could reduce level of ET, but this study may lack large sample to show this association. In future studies, the vague association between rhein and a lower level of ET should be clarified through increasing simple size.

It is of great value to study toxic effects of rhein for clinical rational medication and further research. A study (Hu et al., 2019) indicated that the toxicity of rhein would be obvious in the kidney of mice at the dose of 0.35g/kgċd during intragastric administration which lasted for 60 d, and its mechanisms might be attributable to imbalance of glutathione antioxidant system, inducing excessive oxidation, triggering inflammatory reaction, activating the expression of caspase-3, and inducing apoptosis. Wang (2007) showed that long-term administration of high dose rhein in mice could result in toxic reactions, which were related to the decrease of mitochondrial membrane potential and increase of apoptosis in HK-2 cells and HepG2 cells. He et al. (2014) found that rhein could induce hepatotoxicity in rats, and its toxicological mechanism might be related to the regulation of liver microsomal cytochrome CYP2C19. Based on these researches, it is safe to use rhein under suitable conditions, but hepatotoxicity and nephrotoxicity of rhein would reveal when it was used in long time and at a high dose. In this study, there was no report on the toxic effects of rhein, probably because the dosage and duration of rhein were within a reasonable range. So far, the clinical rational dosage and duration of rhein have not been determined, so it is necessary to focus on this aspect in clinical trials and detect liver and kidney function regularly in the treatment.

DN is caused by the interaction of various factors, and its pathological mechanism is complicated. So, the drug focusing on single target cannot improve the condition of DN. Therefore, drugs with multi-targeting intervention are urgently needed in clinical practice. This meta-analysis showed that rhein had multiple pharmacological effects such as lowering blood glucose, alleviating renal fibrosis, regulating TGF-β_1_, and anti-oxidation, which meets the requirements of clinical medication. So rhein has considerable potential clinical application value. In addition, this meta-analysis could help to optimize clinical trials. It is necessary to include patients with type 1 and type 2 DN as participants, and pay attention to the dosage and duration of rhein. Also, it is essential to consider the combination of rhein and other drugs. For example, rhein could be combined with drugs which are hypoglycemic or alleviate renal fibrosis to determine the optimal treatment program. Nowadays, clinical trials of rhein have been being carried out. A clinical trial indicated that patients with DN were treated by rhein supplementation got better through decreasing Scr, serum cystatin C (CysC), and UAER (Xiao et al., 2011). In addition, the use of rhein capsules has already been approved in Phase II clinical trials (clinical trial approval number: 2008L03643) (Chen et al., 2018). In summary, the clinical trials and animal experiments of rhein need mutual feedback and coordinated development, so that rhein can be applied to the clinical treatment of DN at an early stage.

### Methodological Considerations

Several limitations were needed to be considered in this systematic review and meta-analysis: First, asymmetry of the funnel plots and statistical analysis with Egger’s test indicated that publication bias or small-study effects were in existence, which could exaggerate the effects of intervention. Thus, the positive findings of rhein should be interpreted with caution. Secondly, some included studies did not report baseline of relevant indicators (e.g. urine protein, blood glucose, weight) in the rhein-treated group and the control group. Thirdly, some studies were of poor quality in methodology. For example, the randomized methods of some studies were not clear, which may lead to selective bias. Last, several studies could not be included to conduct meta-analysis due to insufficient data, although these studies claimed that there were positive effects of rhein on outcome measures (e.g. blood glucose, MDA, SOD).

## Conclusion

In summary, this systematic review and meta-analysis suggested that rhein has beneficial effects on animal models of DN, and the mechanisms are positively involved with ameliorating levels of TGF-β_1_, renal fibrosis, metabolism, and oxidative stress status. However, some factors such as possible publication bias, methodological quality, and sample size may affect the accuracy of positive findings. These limitations suggested that a cautious interpretation of the positive results of this systematic review and meta-analysis is necessary. Therefore, high methodological quality and well- reported animal experiments are needed in future research.

## Author Contributions

H-CH, L-TZ, H-YY, and L-PY designed the study. YT searched databases. YT and K-SW collected the data. X-QL and L-TZ assessed the quality of study. H-CH and L-TZ performed all analysis. H-CH, L-TZ, X-QL, and L-PY wrote the manuscript. All authors contributed to this systematic review and Meta-analysis.

## Funding

Funded by the second batch of scientific research projects for the construction of the national TCM clinical research base (No. JDZX2015222).

## Conflict of Interest

The authors declare that the research was conducted in the absence of any commercial or financial relationships that could be construed as a potential conflict of interest.
