# Supplementary material for: A Significant Association Between Rhein and Diabetic Nephropathy in Animals: A Systematic Review and Meta-Analysis
Source: Front Pharmacol. 2019 Dec 13;10:1473. doi: 10.3389/fphar.2019.01473 (PMC6923681; doi:10.3389/fphar.2019.01473)
Supplement: Supplementary file 1 [file DataSheet_1.pdf]

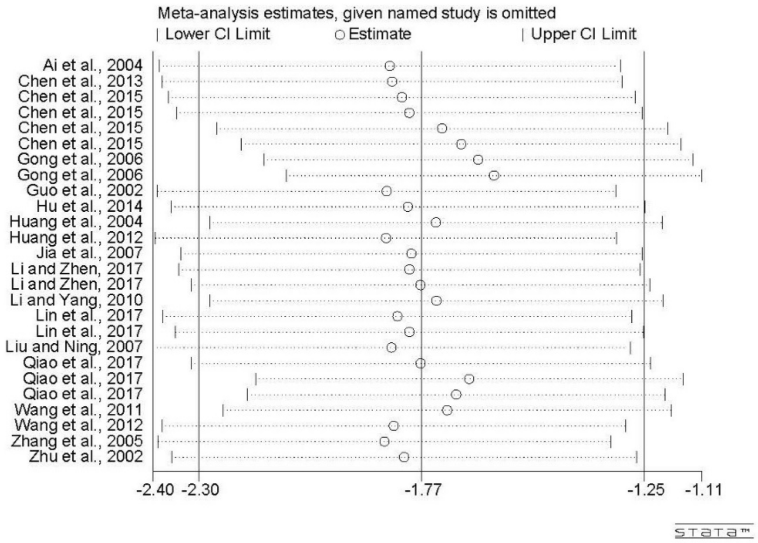

Figure S1 Results of sensitivity analysis according to blood glucose.

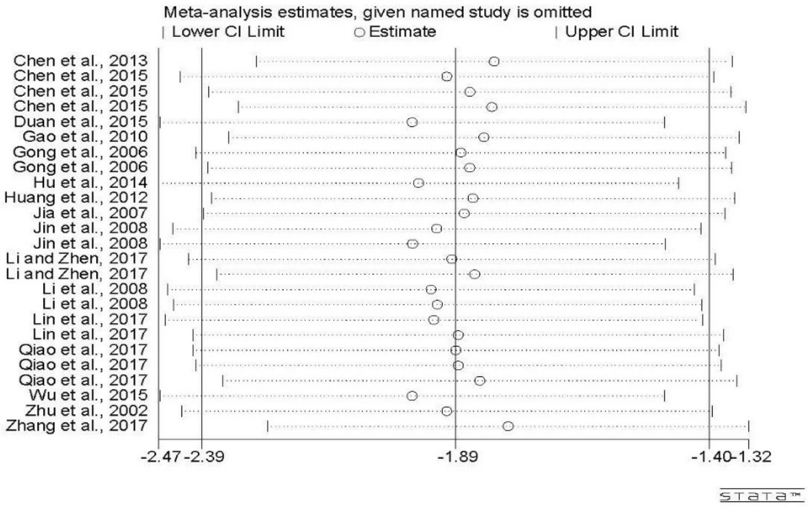

Figure S2 Results of sensitivity analysis according to Scr.

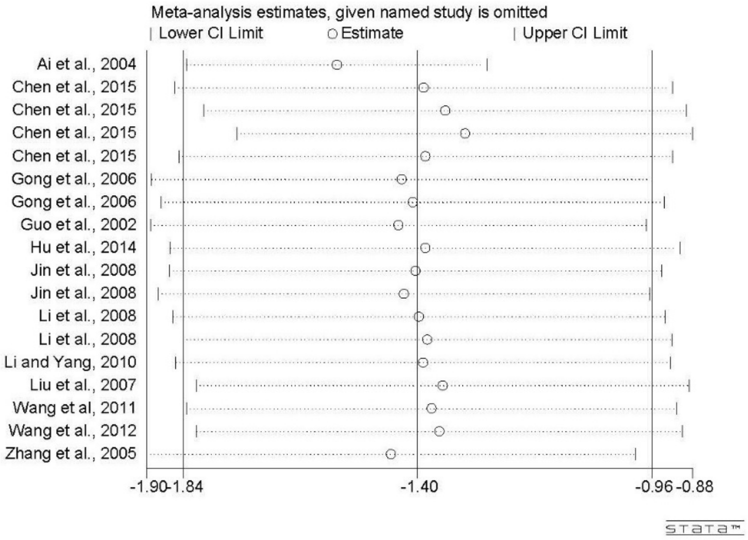

Figure S3 Results of sensitivity analysis according to urine protein.

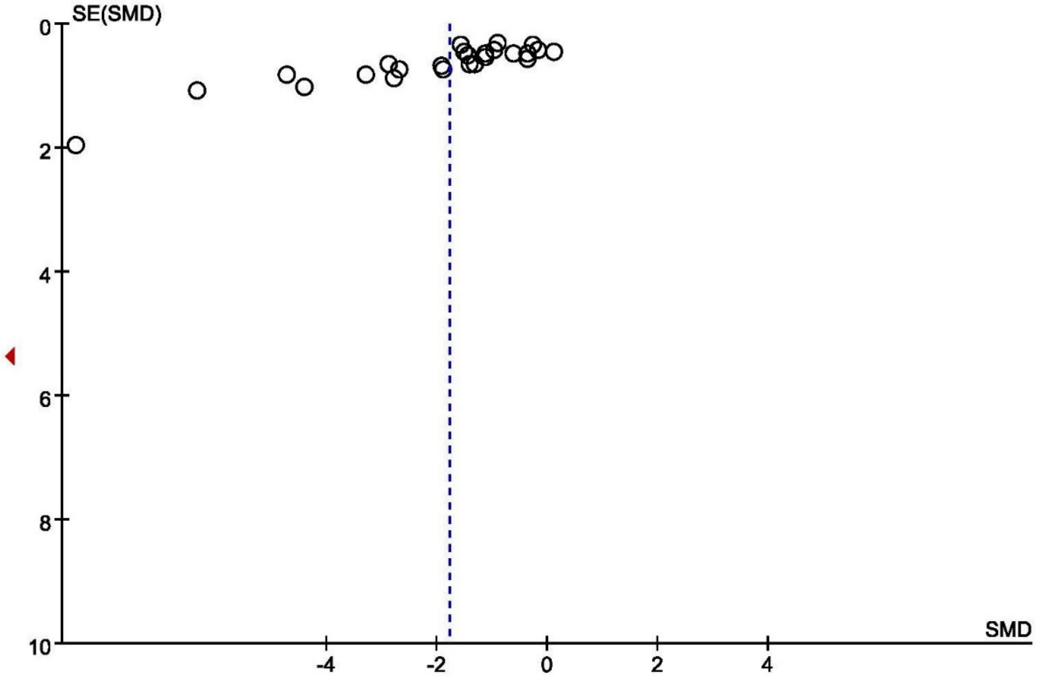

Figure S4 Funnel plot for effectiveness of rhein on blood glucose.

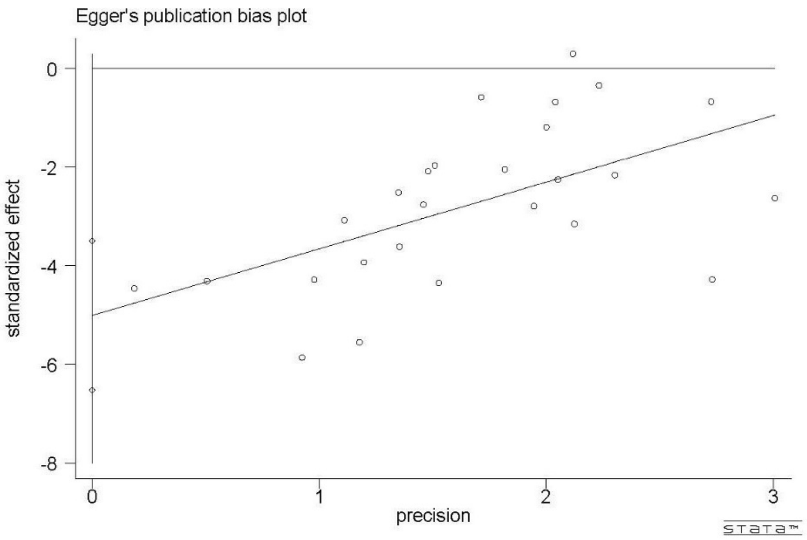

Figure S5 Egger’s test according to blood glucose.

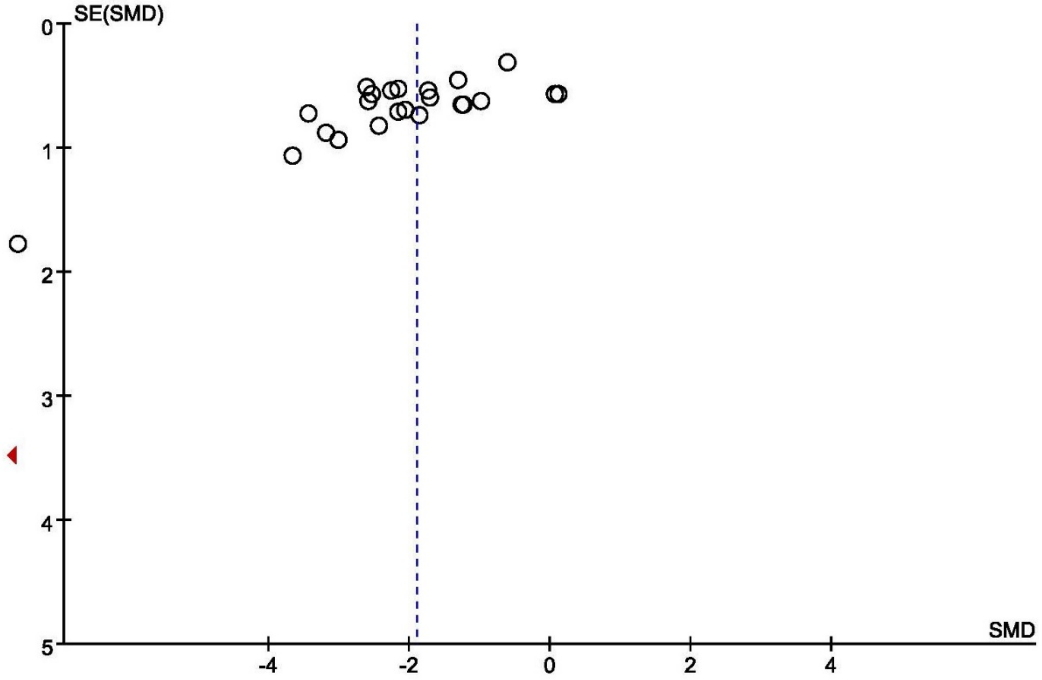

Figure S6 Funnel plot for effectiveness of rhein on Scr.

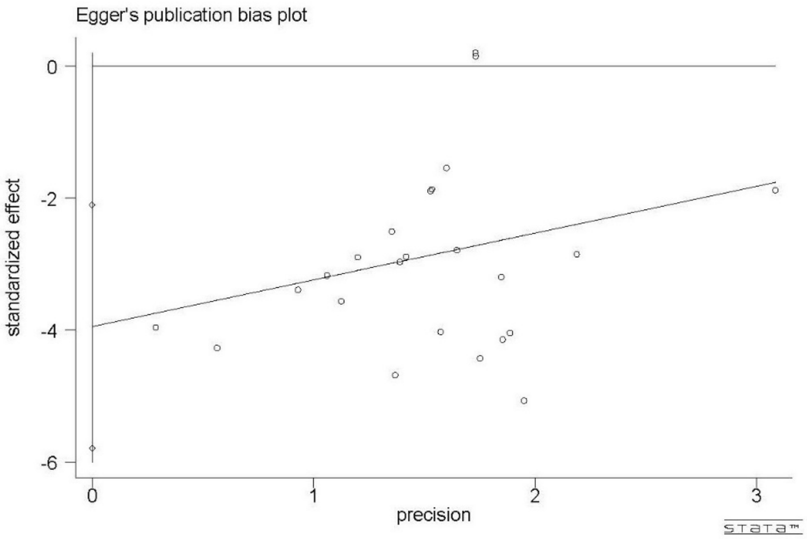

Figure S7 Egger’s test according to Scr.

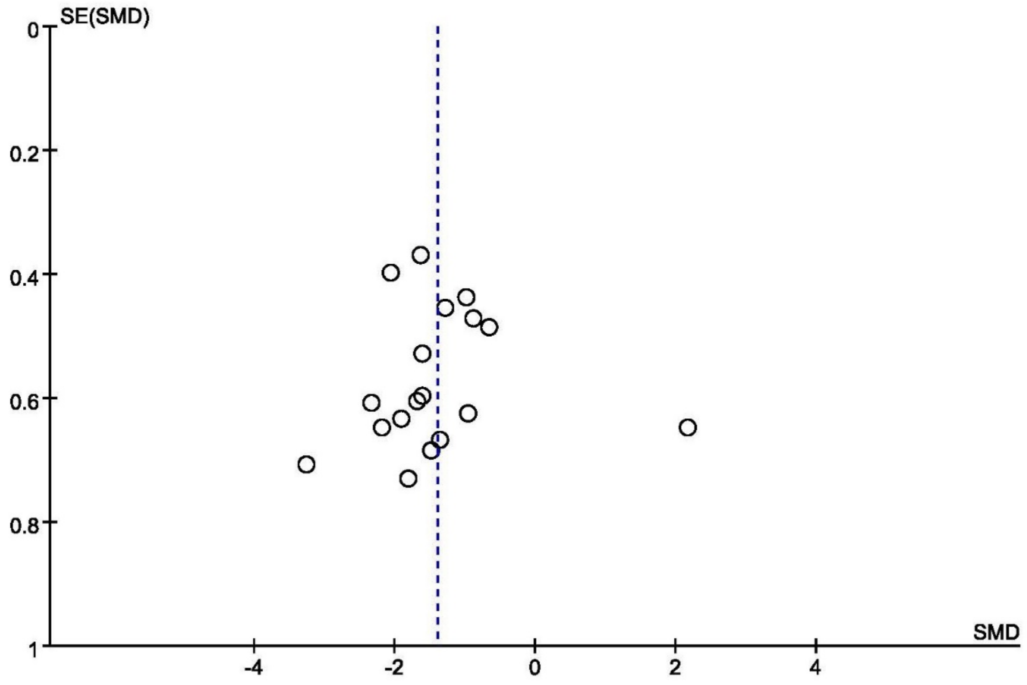

Figure S8 Funnel plot for effectiveness of rhein on urine protein.

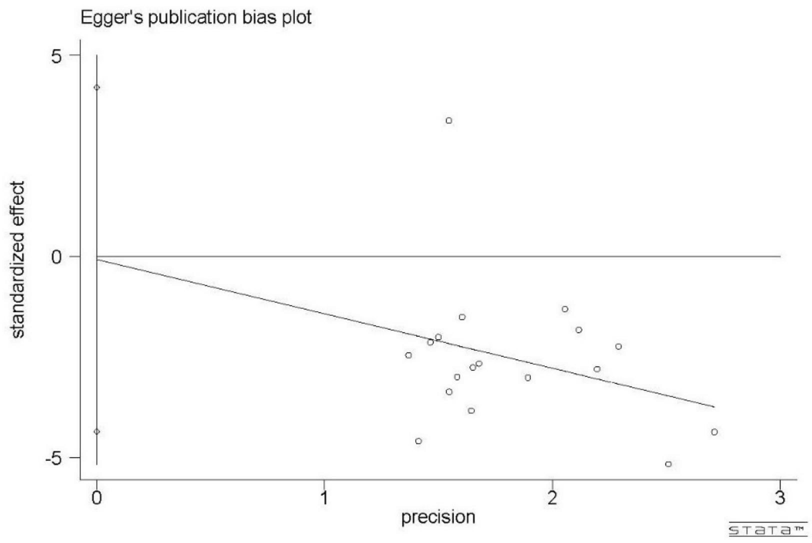

Figure S9 Egger’s test according to urine protein.
