# Supplementary material for: A Significant Association Between Rhein and Diabetic Nephropathy in Animals: A Systematic Review and Meta-Analysis
Source: Front Pharmacol. 2019 Dec 13;10:1473. doi: 10.3389/fphar.2019.01473 (PMC6923681; doi:10.3389/fphar.2019.01473)
Supplement: Supplementary file 2 [file Table_1.docx]

Diabetic nephropathy is one of the most common microvascular complications of diabetes. Patients with diabetic nephropathy are at increased risk of cardiovascular events and mortality. Medical research has shown that diverse pathogenetic mechanisms account for the occurrence of diabetic nephropathy. Because of multiple pathogenic mechanisms, it is likely that multi-targeted therapy will be required for the development of effective therapeutics in diabetic nephropathy. So traditional Chinese herb has received high attention due to its broad pharmacological effects.

Rhein, a monomer extracted from traditional Chinese herb rhubarb. Some clinical trials indicated that rhein has beneficially influence on diabetic nephropathy. However, the related therapeutic mechanisms are still not completely clear, which is one of the main causes of limitation of rhein clinical application. Although some animal experiments supported relevant mechanisms of rhein against diabetic nephropathy, it is difficult to draw definitive conclusions about therapeutic mechanisms due to the small sample size and possible exaggerated efficacy of various individual animal experiments.

Therefore, we conducted systematic review and meta-analysis of pre-clinical animal data. This study would (1) provide more precise estimates to illustrate the efficacy and therapeutic mechanisms of rhein in animal models of diabetic nephropathy, (2) determine the appropriate conditions of rhein to improve the therapeutic effects (optimal dose, proper duration, et al), and (3) determine whether there was an exaggerated effect of rhein by funnel plot and Egger’s test. These questions are essential for rhein further clinical trials and application.
